# Automatic image quality evaluation in digital radiography using a modified version of the IAEA radiography phantom allowing multiple detection tasks: A multi detection task IAEA radiography phantom

**DOI:** 10.1002/acm2.14599

**Published:** 2025-01-13

**Authors:** Ioannis A. Tsalafoutas, Shady AlKhazzam, Virginia Tsapaki, Mohammed Hassan Kharita

**Affiliations:** ^1^ Medical Physics Section, OHS Department Hamad Medical Corporation Doha Qatar; ^2^ NAHU ‐ Dosimetry and Medical Radiation Physics Section IAEA Vienna Austria

**Keywords:** digital radiography, image quality, phantoms, post‐processing

## Abstract

**Purpose:**

To evaluate image quality (IQ) of for‐processing (raw) and for‐presentation (clinical) radiography images, under different exposure conditions and digital image post‐processing algorithms, using a phantom that enables multiple detection tasks.

**Methods:**

A modified version of the radiography phantom described in the IAEA Human Health Series No. 39 publication was constructed, incorporating six additional Aluminum (Al) targets of thicknesses both smaller and larger than the standard one. Raw and clinical images were simultaneously acquired using two digital radiography units from different manufacturers, various exposure parameters and different examination protocols. The phantom images were read using the free IAEA software (ATIA) that estimates automatically various IQ‐metrics in images of the original phantom. Since in the modified phantom had seven Al targets, images were read seven times, one for each different Al thickness. The IQ‐metrics’ values obtained were analyzed to investigate their dependence on incident air kerma on the image receptor, tube potential, examination protocol, image type (raw or clinical), Al‐target thicknesses, and manufacturer.

**Results:**

The IQ‐metric values calculated using the modified IAEA phantom images can be radically different between raw and clinical images, and between different manufacturers, irrespectively whether only the standard or all the different Al‐target thicknesses are considered. The modulation transfer function (MTF) and the signal‐to‐noise‐ratio (SNR) dependence on exposure conditions and post‐processing algorithms do not always follow the same trends for raw and clinical images and/or different manufacturers, while the signal‐difference‐to‐noise‐ratio (SDNR) and the detectability index (d′), despite their differences, seem more appropriate to characterize IQ. However, the d′ metric, which also considers both MTF and the normalized noise power spectrum (NNPS) should be considered more complete IQ‐metric than SDNR.

**Conclusions:**

Though theoretically d′ values should be calculated using raw images, clinical images can be also used, at least for constancy tests.

## 1 INTRODUCTION

Evaluation of image quality (IQ) is an integral part of the QC procedures required for digital radiography (DR) systems.^1^ A typical comprehensive QC procedure is comprised by many QC tests that can be roughly categorized as: (a) technical assessment (checking distances, indications, radiation field and light field alignments, etc.), (b) generator/tube tests and dosimetry measurements, (c) automatic exposure control (AEC) system tests and finally (d) IQ tests. Such QC procedures are time‐consuming and for this reason are typically performed annually. However, when it comes to constancy tests, there are simple and fast QC tests that can be performed on a more frequent basis (weekly or even daily), to give a reliable overview of the operation of the whole imaging chain.^1^ **^,^** ^2^

A simple option is to acquire a flat field image of the whole image receptor using a uniform attenuator (e.g., 2 mm Cu) under AEC, which can assess: the uniformity of the image receptor (visual review for non‐uniformities or artifacts that can be due to the image receptor, table or grid), the accuracy and consistency of the exposure index (EI) indicator and the consistency of AEC operation and generator [tube loading (mAs)]. Using a radiation field ∼2 cm smaller than the image receptor size will also allow to check the alignment of light and radiation fields, at the cost of not checking the uniformity of 2 cm frame on the edges of the image receptor. Any drift of one or more parameters from the previous QC's values cannot always pinpoint which is the link in the chain that has failed and may call for a repeat of parts or the whole comprehensive QC procedure. For example, if the real kVp has increased with respect to the nominal value (let's say 85 kV instead of 80 kV), the mAs value will be reduced, but this may also happen if the target incident air kerma (IAK) of the AEC has drifted downwards. In the first case however, EI will not be reduced whereas in the second it will. However, as long as the mAs and EI remain constant, this indicates that tube potential (kVp), tube output, AEC target IAK, also referred to as switch‐off dose, have not changed. Of course, a uniform phantom, cannot provide further information regarding IQ, and a second exposure of a physical phantom typically containing various structures to assess spatial resolution, low contrast, dynamic contrast, and so forth. will be required to do this, either using visual/manual methods or appropriate software.^3^

Based on the above concepts, the International Atomic Energy Agency (IAEA) has developed a remote and automated solution to perform constancy QC tests, that allows for a complete and automated evaluation of the principal performance characteristics of the imaging chain, using a single image of a simple and inexpensive homemade phantom, and free software called Automated Tool for Image Analysis (ATIA).^2^ According to this methodology, the IQ is evaluated using traditional metrics like signal‐to‐noise‐ratio (SNR), signal‐difference‐to‐noise‐ratio (SDNR), and modulation transfer function (MTF), but also the detectability index (d′). The d’ is an IQ‐metric that simulates the human observer performance in interpretation tasks like the detectability of contrast‐detail test objects of certain size and shape and thus it can be directly linked to clinical imaging performance.^2^ **^,^** ^4^ However, its accuracy has been validated in raw images only.^5^ **^,^** ^6^

To validate this methodology IAEA has launched a coordinated research project (CRP) entitled “Advanced Tools for Quality and Dosimetry of Digital Imaging in Radiology”, to which many DR and mammography facilities around the world were initially enlisted and more are gradually getting on‐board.^7^ **^‐^** ^9^ According to the IAEA methodology, the use of for‐processing images (henceforth referred to as **raw**), is preferred for IQ evaluation over for‐presentation (henceforth referred to as **clinical**) images for both radiography and mammography.^2^ However, when raw images are not produced by the x‐ray system or are not easy to access, the use of clinical images is proposed alternatively. In this case the use of the Abdomen protocol is suggested, which however should be consistently used for all subsequent constancy tests.^2^

In two previously published studies, the effect that a change in the quality and/or the intensity of the x‐ray beam may have on the IQ metrics calculated by ATIA was investigated, in clinical images from a DR system from a single manufacturer^3^ and in both raw and clinical images from another DR manufacturer.^10^ When comparing the results of these two studies,^3^ **^,^** ^10^ differences between manufacturers were observed regarding the dependence of some IQ metrics on certain exposure parameters and the respective trends. However, what was rather unexpected, is that d′ was consistently larger in raw images than in the respective clinical images.^10^ It was postulated that this may be attributed to the fact that clinical images use post‐processing algorithms which are intended to optimize the visual detection of the anatomy of interest (commonly found within a complex background) by human observers and not software. It was also thought that d′ is calculated under specific conditions (80 kVp) for a specific interpretation task (detection of conceptual holes of diameters 0.3 and 4 mm of contrast equal to that of the 4 mm Al square in a uniform background of 2 mm Cu and 0.5 mm PMMA), which is not representative of the different detection tasks encountered in the clinical practice. Images of human anatomy contain more than one anatomical structure (targets) with different contrast levels (pixel values [PV] and SDNR differences) from the surrounding structures or tissues (background), and at various brightness (exposure) levels. The post‐processing algorithm considers that all anatomical structures of interest, must be simultaneously visualized by radiologists, with or without manual adjustments of window width and window level to better visualize certain parts of the image. Therefore, it is questionable whether for other detection tasks d’ values would be always bigger in raw images.

In this study, to investigate the IQ‐metrics’ differences between raw and clinical images for different detection tasks, the IAEA phantom was modified to include more Al targets and the methodology used in the two referenced studies^3^ **^,^** ^10^ was repeated using both raw and clinical images from two DR systems of different manufacturers.

## 2 MATERIALS AND METHODS

**FIGURE 1:**
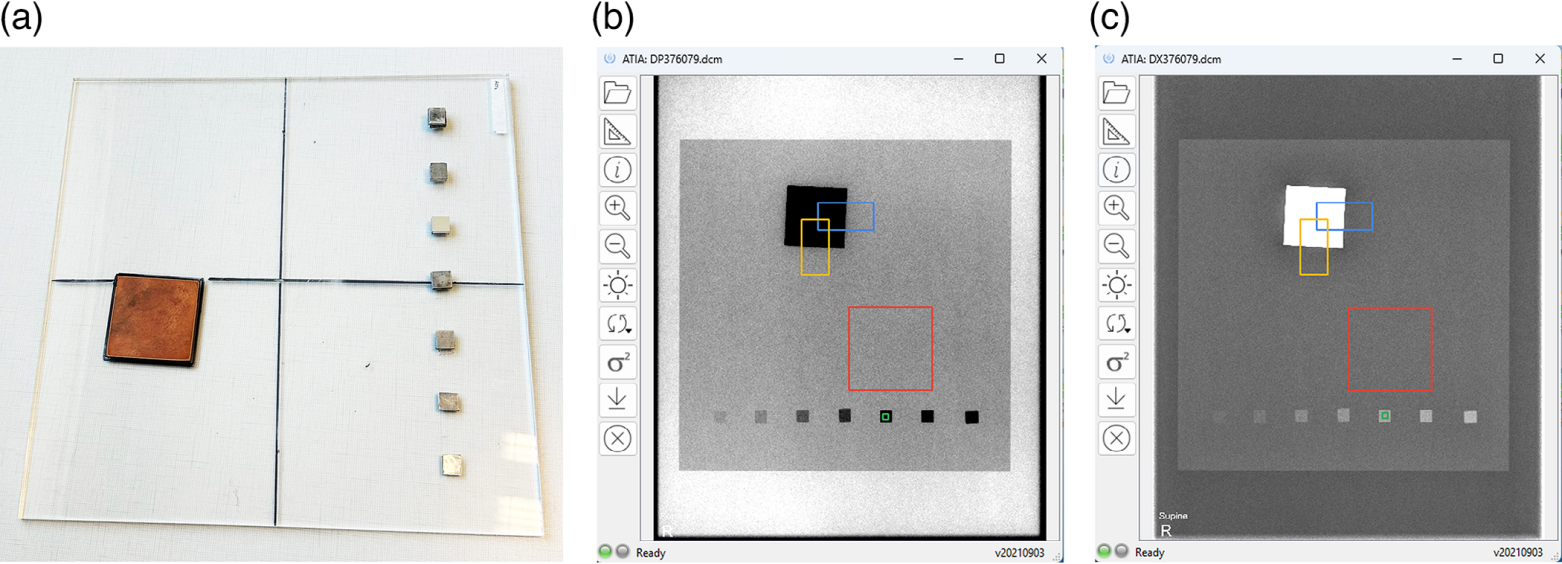
The IAEA modified radiography phantom (Multi): (a) Photograph, (b) Radiographic appearance of the Multi phantom within the ATIA software (Philips clinical image). (c) Radiographic appearance of the Multi phantom within the ATIA software (Philips raw image). Note that a 2 mm Cu sheet is also positioned on the *x*‐ray tube as attenuator. The orange, blue, red, and green ROIs are automatically positioned by the software to calculate the IQ metrics. IAEA, International Atomic Energy Agency; IQ, image quality; ROI, regions of interest.

The modified IAEA phantom (henceforth, referred to as **Multi**) is shown in Figure 1. The only difference from the original IAEA radiography phantom (henceforth referred to as the IAEA phantom) is that it has seven aluminum (Al) targets of 0.5‐, 1‐, 2‐, 3‐, 4‐ 5‐ and 6‐mm thickness, whereas the IAEA phantom has only one Al target (4 mm).^2^ **^,^** ^10^ Thus, the Multi phantom can simulate additional detection tasks, both more difficult (i.e., details of less contrast, represented by the Al targets with thicknesses smaller than 4 mm) and less difficult (i.e., details of more contrast, represented by the Al target thicknesses larger than 4 mm) than the standard one in the original IAEA phantom. The different Al target thicknesses serve as a step‐wedge that can be found in some commercial QC phantoms.^3^

The images of the original IAEA phantom are automatically evaluated using the ATIA software which calculates the following IQ metrics: spatial resolution (MTF @ 50%, 20%, and 10% in the horizontal and vertical directions), SNR, SDNR, d′ (*D* = 0.3 mm) and d′ (*D* = 4 mm).^2^ **^,^** ^4^ To evaluate an image of the multi phantom, the ATIA must be run seven times, one for each different Al square thickness, with manual re‐positioning of the green ROI shown in Figure 1b,c, while the positions of the blue, orange, and red ROIs remain constant. Thus, seven results files are generated for each image, all having identical MTF values, but different SNR, SDNR, and d′ values, due to the different Al square thicknesses and the different positions of the green ROI. Note that the background signal and noise is determined using four small ROIs (not shown by ATIA) around the respective green ROI, and this is why SNR changes though not directly affected by the different Al thicknesses.

The DRs units used to acquire the Multi phantom images were a General Electric (GE) Discovery XR656HD (GE HealthCare, Chicago, Illinois, USA) and a Philips x‐ray system (Digital Diagnost, Philips Healthcare, Best, Netherlands). The first system routinely produces a raw and a clinical image for each exposure, while the second only a clinical image. However, in answer to our request, the Philips system was adjusted by the field service engineers to send both clinical and raw images to the Medical Physicists’ workstation. Both DR systems had been subjected to an extensive quality control (QC) testing prior to the following experiments, and all parameters including kVp accuracy, kVp, IAK, and AEC system reproducibility, were found to be well within the adopted performance limits.^11^

All images (raw and clinical) were acquired in one go, with the Multi phantom positioned on the radiography table of each unit, without moving the phantom or changing the radiation field size. The basic examination protocol used was the Abdomen AP, with the kVp adjusted to 80 for GE and 81 kV for Philips, with the AEC system activated (central AEC cell only), the antiscatter grid in place and without additional filtration on the tube. Note that the AEC of both systems are adjusted to terminate the exposure for an IAK (switch‐off dose) of about 2.5 µGy on the image receptor. Four different experiments were performed as described in the following.

***Experiment 1***: Repeated acquisitions were carried out using the basic examination protocol, to determine the range of variations expected in the IQ‐scores of the Multi phantom images resulting from repeated acquisitions with identical exposure settings (due to Poisson statistics and minor drifts that may occur during repeated exposures under AEC).
***Experiment 2***: Acquisitions were carried out using different examination protocols (Abdomen AP, Chest AP/PA, Ribs AP, Thoracic Spine AP, Pelvis AP, and Skull LL/Routine Skull AP). These examination protocols use different preset post‐processing algorithms, which have been designed to optimize the IQ for the relevant anatomical area. The preset kVp and AEC settings of these examination protocols were switched to those of the basic acquisition protocol (80/81 kV and central AEC sensor activated only), so that the distinct effect of the post‐processing algorithms on the IQ‐scores of the Multi phantom could be discerned.
***Experiment 3***: Acquisitions were made with the basic acquisition protocol, except that IAK on the image receptor was varied (manually in the GE system and using AEC dose level correction settings of −2.5, −1.5, −1, 0, +1, +1.5, +2.5 in the Philips system). The purpose of this experiment was to determine how the increase of IAK on the image receptor affects the IQ‐scores.
***Experiment 4***: Acquisitions were made with the basic acquisition protocol, except that the kVp was varied from 60 to about 120 kV, to determine the effect of increasing kVp on the IQ‐scores.

The acquisition and processing parameters of all phantom images were obtained from their DICOM headers, using a free software named DICOM Info Extractor.^12^

## 3 RESULTS

**FIGURE 2:**
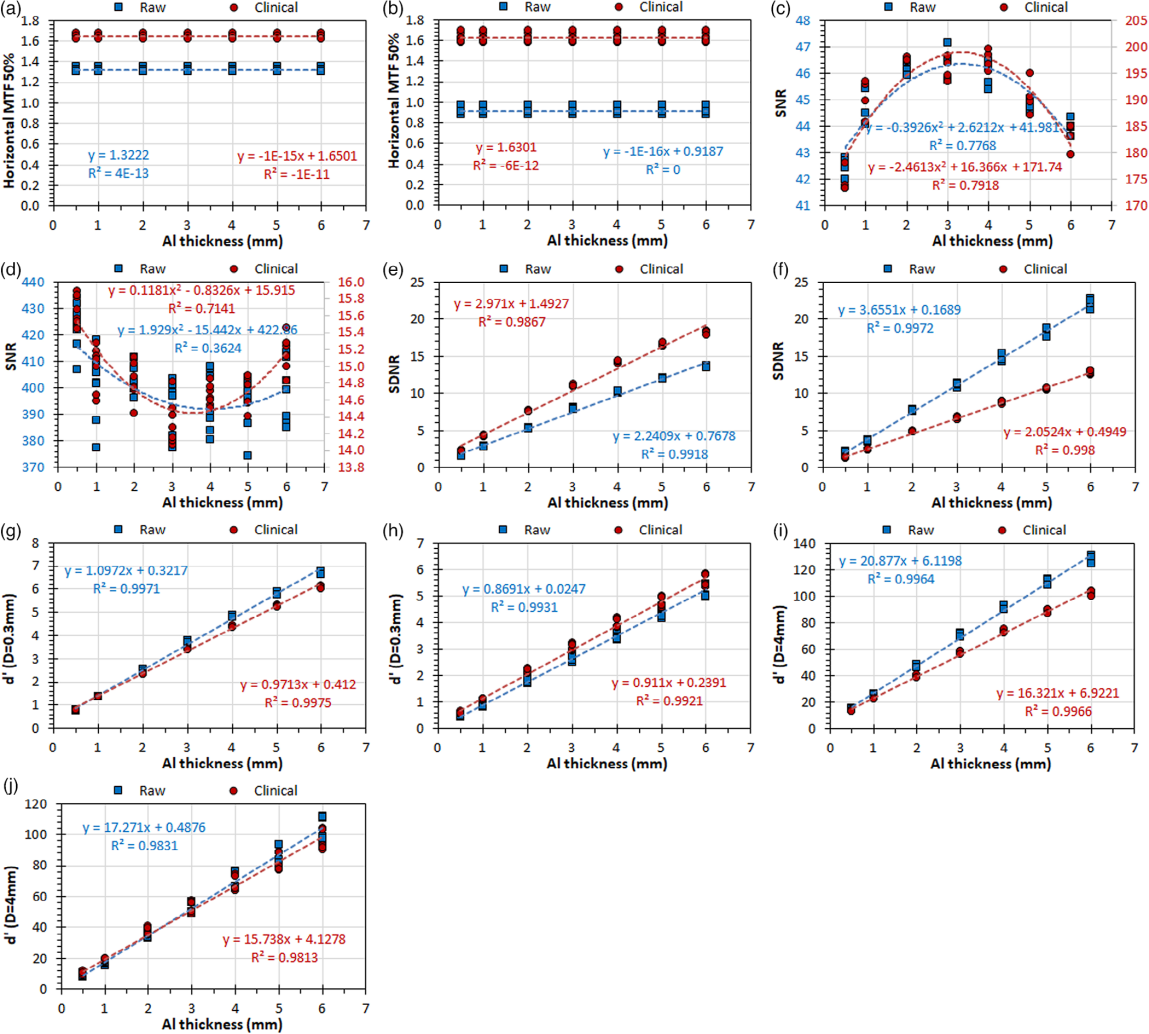
The variation of IQ metrics with the Al target thickness in four repeated acquisitions with the Abdomen AP protocol, for raw and clinical images of the Multi phantom: (a) GE: MTF 50%, (b) Philips: MTF 50%, (c) GE: SNR, (d) Philips: SNR, (e) GE: SDNR, (f) Philips: SDNR, (g) GE: d′ (*D* = 0.3 mm), (h) GE: d′ (*D* = 0.3 mm), (i) GE: d′ (*D* = 4 mm), (j) Philips: d′ (*D* = 4 mm). For the images (c) and (d), where two *Y*‐axes exist, the left *Y*‐axis (blue values) corresponds to the blue data points (raw), while the right *Y*‐axis (dark‐red values) corresponds to the dark‐red data points (clinical). GE, general electric; MTF, modulation transfer function; SDNR, signal‐difference‐to‐noise‐ratio; SNR, signal‐to‐noise‐ratio.

**FIGURE 3:**
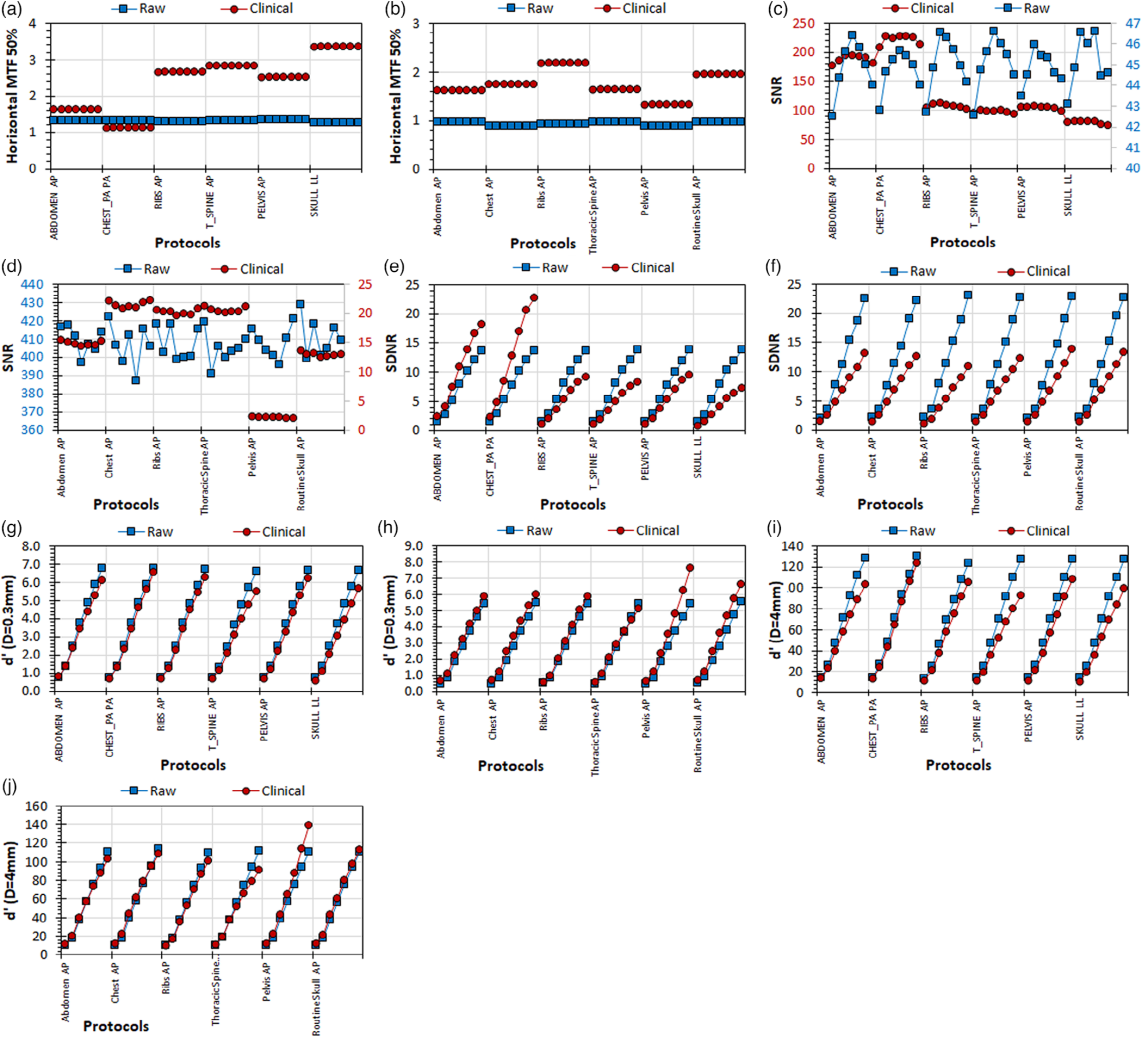
The variation of the IQ metrics with the Al target thickness with six different protocols, for raw and clinical images of the Multi phantom: (a) GE: MTF 50%, (b) Philips: MTF 50%, (c) GE: SNR, (d) Philips: SNR, (e) GE: SDNR, (f) Philips: SDNR, (g) GE: d′ (*D* = 0.3 mm), (h) GE: d′ (*D* = 0.3 mm), (i) GE: d′ (*D* = 4 mm), (j) Philips: d′ (*D* = 4 mm). For each protocol, the Al thickness increases from left to right, starting from 0.5 mm. For the images c and d, where two *Y*‐axes exist, the left *Y*‐axis (blue values) corresponds to the blue data points (raw), while the right *Y*‐axis (dark‐red values) corresponds to the dark‐red data points (clinical). GE, general electric; MTF, modulation transfer function; SDNR, signal‐difference‐to‐noise‐ratio; SNR, signal‐to‐noise‐ratio.

**FIGURE 4:**
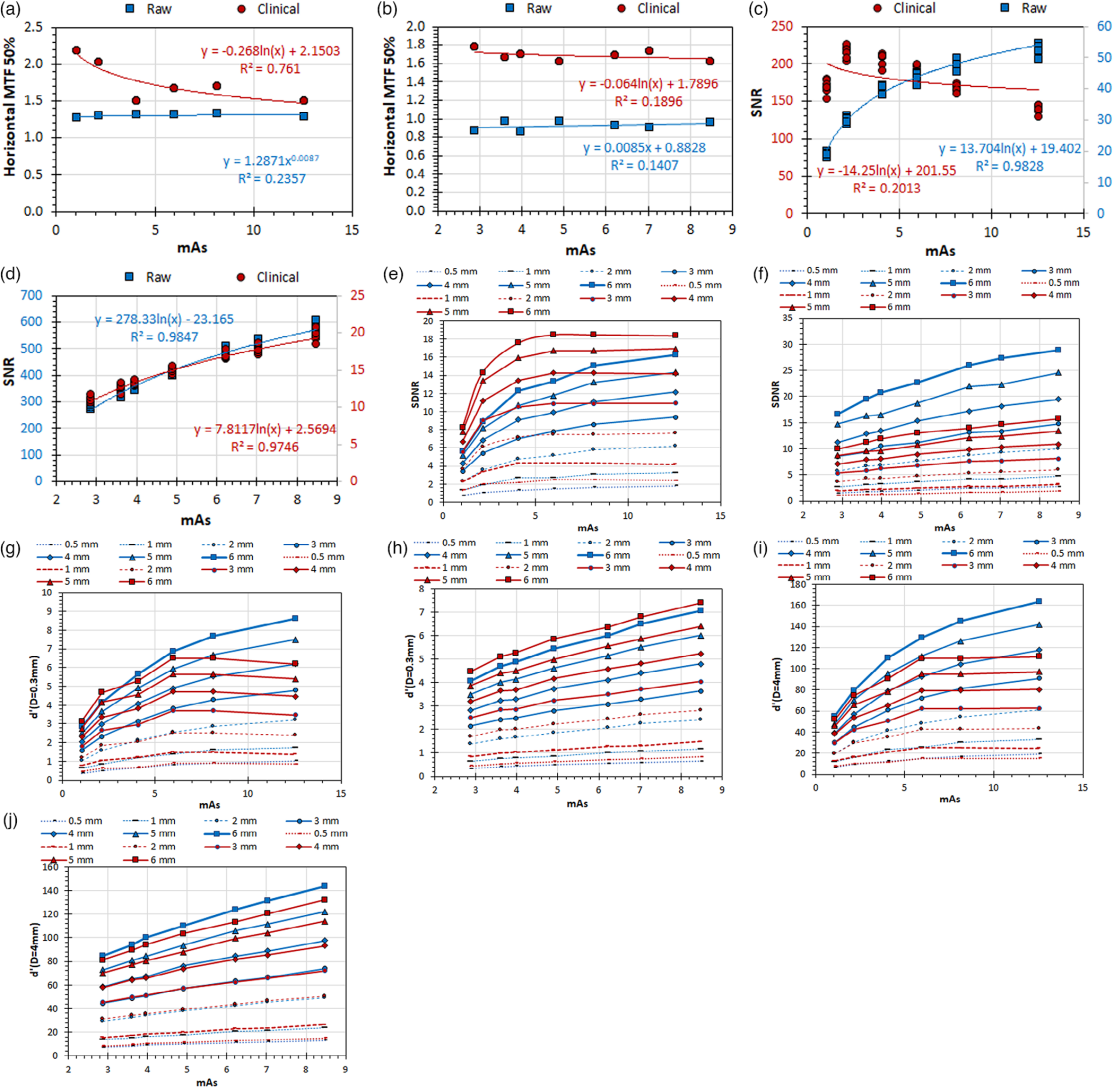
The variation of the IQ metrics with the Al target thickness with different mAs values, for raw and clinical images of the Multi phantom: (a) GE: MTF 50%, (b) Philips: MTF 50%, (c) GE: SNR, (d) Philips: SNR, (e) GE: SDNR, (f) Philips: SDNR, (g) GE: d′ (*D* = 0.3 mm), (h) GE: d′ (*D* = 0.3 mm), (i) GE: d′ (*D* = 4 mm), (j) Philips: d′ (*D* = 4 mm). The mAs values for AEC with 0 dose level selection were 6 mAs for GE and 4.9 mAs for Philips. For the images (c) and (d), where two *Y*‐axes exist, the left *Y*‐axis (blue values) corresponds to the blue data points (raw), while the right *Y*‐axis (dark‐red values) corresponds to the dark‐red data points (clinical). AEC, automatic exposure control; GE, general electric; MTF, modulation transfer function; SDNR, signal‐difference‐to‐noise‐ratio; SNR, signal‐to‐noise‐ratio.

**FIGURE 5:**
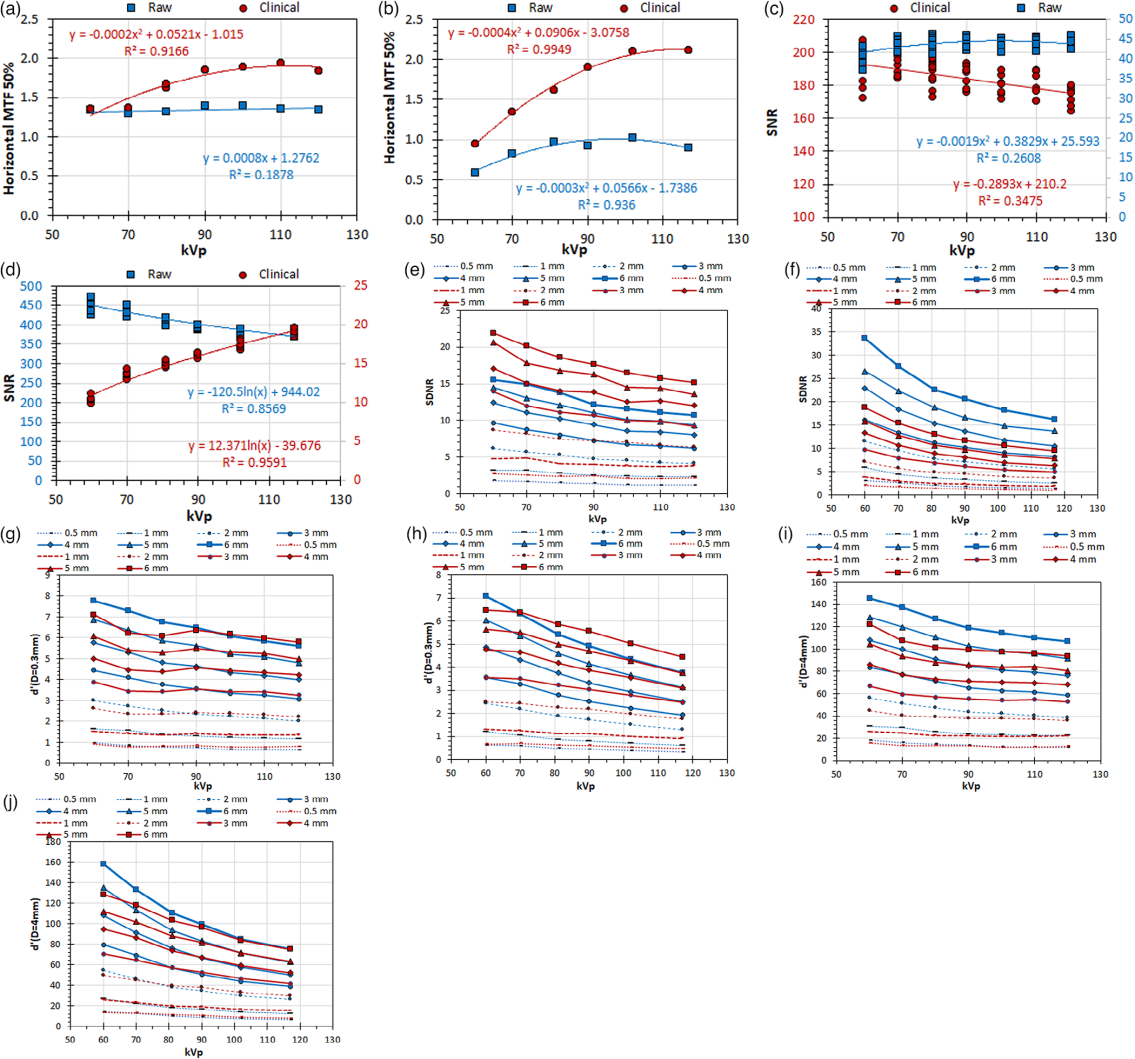
The variation of the IQ metrics with the Al target thickness with different kVp values, for raw and clinical images of the Multi phantom acquired with a GE and a Philips system: (a) GE: MTF 50%, (b) Philips: MTF 50%, (c) GE: SNR, (d) Philips: SNR, (e) GE: SDNR, (f) Philips: SDNR, (g) GE: d′ (*D* = 0.3 mm), (h) GE: d′ (*D* = 0.3 mm), (i) GE: d′ (*D* = 4 mm), (j) Philips: d′ (*D* = 4 mm). For the images (c) and (d), where two *Y*‐axes exist, the left *Y*‐axis (blue values) corresponds to the blue data points (raw), while the right *Y*‐axis (dark‐red values) corresponds to the dark‐red data points (clinical). GE, general electric; MTF, modulation transfer function; SDNR, signal‐difference‐to‐noise‐ratio; SNR, signal‐to‐noise‐ratio.

The main results of this study regarding the use of the Multi phantom as the original IAEA radiography phantom are tabulated in Table 1 (blocks 1−4), whereas the detailed analysis of the reported MTF, SNR, SDNR, and d′ values and their variations with different exposure conditions and different Al thicknesses are presented in Figures 2, 3, 4, 5.

**TABLE 1:** Blocks 1−4: Summary of the IQ evaluation results considering the 4 mm Al target only.

Experiment		1. Repeated acquisitions (Poisson statistics)				2. Changing post‐processing algorithm				3. Increasing IAK on image receptor				4. Increasing kVp			
IQ metrics	Statistical parameter	Raw GE	Clin. GE	Raw Philips	Clin. Philips	Raw GE	Clin. GE	Raw Philips	Clin. Philips	Raw GE	Clin. GE	Raw Philips	Clin. Philips	Raw GE	Clin. GE	Raw Philips	Clin. Philips
Horizontal MTF 50%	min	1.30	1.62	0.88	1.58	1.27	1.14	0.88	1.33	1.27	1.50	0.86	1.62	1.30	1.36	0.59	0.95
	max	1.35	1.68	0.91	1.67	1.34	3.34	0.98	2.19	1.33	2.18	0.98	1.78	1.40	1.95	1.03	2.11
	Mean	1.32	**1.65**	0.89	**1.62**	1.31	**2.35**	0.94	**1.75**	1.30	**1.76**	0.93	**1.69**	1.35	**1.70**	0.87	**1.67**
	max/min	**1.04**	1.04	**1.04**	1.05	**1.06**	2.94	**1.12**	1.65	**1.04**	1.45	1.13	**1.10**	**1.08**	1.44	**1.74**	2.21
Vertical MTF 50%	Min	1.34	1.66	0.87	1.57	1.33	1.17	0.87	1.36	1.32	1.55	0.87	1.46	1.30	1.44	0.64	0.90
	Max	1.41	1.71	0.95	1.69	1.38	4.04	0.98	2.24	1.42	2.40	1.00	1.70	1.56	2.13	0.99	2.12
	Mean	1.37	**1.70**	0.93	**1.64**	1.36	**2.70**	0.94	**1.76**	1.37	**1.89**	0.95	**1.64**	1.44	**1.85**	0.87	**1.64**
	max/min	1.05	**1.04**	1.08	**1.08**	**1.04**	3.45	**1.13**	1.65	**1.07**	1.55	**1.15**	1.17	**1.20**	1.48	**1.55**	2.36
SNR	Min	45.40	195.3	14.27	8.55	45.3	80.4	14.4	7.30	19.17	145.3	11.22	7.09	42.80	179.0	10.5	6.36
	Max	46.47	199.7	14.77	8.77	46.6	227.0	15.4	9.16	54.67	222.1	19.47	10.91	46.20	197.6	22.9	13.31
	Mean	45.92	**197.5**	**14.52**	8.68	45.8	**135.5**	**15.0**	8.65	39.97	**185.3**	**15.39**	9.01	44.91	**189.4**	**15.4**	9.04
	max/min	1.02	**1.02**	1.04	**1.03**	**1.03**	2.82	**1.07**	1.25	2.85	**1.53**	1.74	**1.54**	**1.08**	1.10	2.19	**2.09**
SDNR	Min	10.10	14.05	380.4	14.54	10.07	5.44	387.1	2.12	4.29	6.69	270.6	10.99	8.05	12.02	369.4	10.04
	Max	10.31	14.40	390.1	14.85	10.4	17.00	407.2	20.93	12.15	14.42	571.8	19.21	12.40	17.10	456.8	18.81
	Mean	10.22	**14.24**	**385.7**	14.68	**10.22**	9.46	**399.7**	15.08	8.89	**12.36**	**416.8**	14.91	9.74	**13.90**	**406.4**	14.88
	max/min	**1.02**	1.02	1.03	**1.02**	**1.03**	3.12	**1.05**	9.89	2.83	**2.15**	2.11	**1.75**	1.54	**1.42**	**1.24**	1.87
Detectability Index d′ (*D* = 0.3 mm)	Min	4.78	4.37	3.37	3.80	4.76	3.94	3.67	3.70	2.05	2.30	2.81	3.20	4.01	4.22	2.51	3.11
	Max	4.88	4.43	3.42	3.86	4.88	4.58	3.78	4.80	6.20	4.72	4.81	5.23	5.77	5.00	4.86	4.77
	Mean	**4.84**	4.40	3.40	**3.83**	**4.82**	4.27	3.73	**4.31**	**4.29**	3.87	3.77	**4.19**	**4.72**	4.49	3.61	**4.03**
	max/min	1.02	**1.01**	**1.02**	1.02	**1.03**	1.16	**1.03**	1.30	3.03	**2.05**	1.71	**1.64**	1.44	**1.18**	1.94	**1.53**
Detectability Index d′ (*D* = 4 mm)	Min	90.1	72.35	65.3	63.86	89.1	67.09	75.10	66.14	39.0	38.3	58.6	57.97	76.4	68.08	50.25	52.45
	Max	93.1	75.27	66.9	65.29	93.6	86.60	77.40	88.0	117.5	80.5	97.7	93.28	108.1	86.08	108.3	94.75
	Mean	**92.1**	73.90	**66.0**	64.60	**91.5**	74.47	75.79	**76.2**	**81.6**	65.4	**76.9**	74.64	**88.6**	73.59	**75.00**	72.26
	max/min	**1.03**	1.04	1.03	**1.02**	**1.05**	1.29	**1.03**	1.33	3.01	**2.10**	1.67	**1.61**	1.41	**1.26**	2.15	**1.81**

*Note*: The max/min ratio is the ratio of the maximum (max) and the minimum (min) values of the IQ metric value observed in each experiment, and is used to denote whether there is an effect of the tested parameter on the respective IQ metric. Regarding the raw versus clinical image IQ‐scores comparisons, with bold are given the mean values of the IQ metrics which are bigger, and the max/min ratio values which are smaller.

Abbreviations: IAK, incident air kerma; IQ, image quality; MTF, modulation transfer function; SDNR, signal‐difference‐to‐noise‐ratio; SNR, signal‐to‐noise‐ratio.

***Experiment 1***: The first difference observed between GE and Philips system is related to the appearance of raw images. GE raw images had similar appearance as the clinical images (Cu was brighter than PMMA background, as in Figure 1b), whereas the Philips raw images were inverted compared to the clinical images (Cu was darker than PMMA background, as in Figure 1c). However, for both GE and Philips, the PV of Cu compared to PV of PMMA were smaller for raw images, and larger for clinical images.

Regarding the maximum variation observed in IQ‐scores with repeated identical exposures (Table 1, Block 1), the variation in mAs using AEC when keeping all exposure and geometrical parameters constant was minimal (< 3% for GE and < 1% for Philips). The maximum variation observed in all IQ‐scores was 4% (largest max/min ratio = 1.04) for GE and 8% for Philips (largest max/min ratio = 1.08).

For the GE system the MTF 50% values are slightly larger (∼25%) for clinical images (Figure 2a), whereas for the Philips system the MTF 50% values for clinical images are roughly double that of raw images (Figure 2b). For GE the SNR values for clinical images are about four times larger than the respective SNR values for clinical images (Figure 2c), whereas for Philips the SNR values for raw images are about 30 times the values of clinical images (Figure 2d). For GE the SDNR values are larger for clinical images (Figure 2e), whereas for Philips the SDNR values are larger for raw images (Figure 2f). For GE the d′ (*D* = 0.3 mm) values are larger for raw images, though for 0.5‐ and 1‐mm Al thicknesses are practically the same (Figure 2g), whereas for Philips the d′ (*D* = 0.3 mm) values are always larger for clinical images (Figure 2h). For GE the d′ (*D* = 4 mm) values are larger for raw images, though they tend to converge for small Al thicknesses (Figure 2i). For Philips the d′ (*D* = 4 mm) values for raw images are slightly larger than those for clinical images for large aluminum thicknesses, but as Al thickness is reduced the reverse is true (Figure 2j).

Regarding the variation with different Al thickness, for GE the SNR values are larger for medium thickness (2–3 mm Al), whereas for Philips the SNR values for smaller and larger thicknesses are slightly larger than those for medium thicknesses. The SDNR, d′ (*D* = 0.3 mm) and d′ (*D* = 4 mm) values increase linearly with increasing Al thickness for both systems (correlation coefficient *R* ^2 ^= 1).

The results of this experiment alone highlight some distinct differences in the behavior of imaging systems of different manufacturers regarding the relative magnitude of some IQ‐metrics in raw and clinical phantom images (e.g., MTF 50%, SNR and SDNR). However, as expected, for both systems SDNR and d’ values increase with Al target thickness in both raw and clinical phantom images.

***Experiment 2***: Regarding the effect of post‐processing algorithm (Table 1, Block 2), it was observed that the maximum variations observed in IQ‐scores with different examination protocols were much smaller in raw images (the largest max/min ratio is equal to 1.06 for GE and 1.13 for Philips for MTF 50%) than those observed in the clinical images (largest max/min ratio = 3.45 for GE and 9.89 for Philips).

For the GE system the MTF 50% values are larger for clinical images for all protocols tested except for Chest PA (Figure 3a), whereas for the Philips system the MTF 50% values are larger for clinical images for all protocols tested (Figure 3b). The SNR values are always much larger for the clinical images for the GE system and for the raw images for the Philips system (Figure 3c,d). For GE the SDNR values are larger for clinical or raw images, depending on the protocol (Figure 3e), whereas for Philips the SDNR values were always larger for raw images (Figures 3f). For GE the d′ (*D* = 0.3 mm) values are larger for raw images but for smaller Al thicknesses are practically the same for raw and clinical images (Figures 3g), whereas for Philips the d′ (*D* = 0.3 mm) values for all protocols are slightly larger for clinical images (Figure 3h). For GE the d′ (*D* = 4 mm) values are always larger for raw images (Figure 3i), whereas for Philips the d′ (*D* = 4 mm) a mixed behavior was observed depending on the protocol and the Al thickness (Figure 3j).

The results of this experiment highlight the variable impact of different post‐processing algorithms on IQ‐scores. Differences are pronounced in clinical images for both GE and Philips, in contrast to raw images where differences are minimal. For all examination protocols and for both GE and Philips systems, the SDNR and d’ values reduce with reducing Al‐target thickness, despite differences that may exist between raw and clinical phantom images regarding the relative magnitude of these metrics. However, for some protocols the d’ values for raw and clinical images are practically equal for all Al‐thickness.

***Experiment 3***: Regarding the effect of increasing mAs (surrogate for IAK) on the IQ‐scores (reported in Table 1, Block 3), for both GE and Philips systems, the MTF 50% values are larger for clinical images and do not change with increasing mAs, except for the clinical images acquired with the GE system where MTF 50% values reduce with increasing mAs (Figure 4a,b). For GE the SNR values are larger for clinical images (Figure 4c), whereas for Philips are larger for raw images (Figure 4d). The SNR values increase with increasing mAs, except for the clinical images acquired with the GE system where above 2 mAs reduce with increasing mAs. For GE the SDNR values are larger for clinical images (Figure 4e), whereas for Philips the SDNR values are larger for raw images (Figure 4f). For both GE and Philips, the SDNR values increase continuously with mAs, but for the clinical images acquired with GE, a plateau is reached at the mAs value selected by the AEC.

For the GE system the d′ (*D* = 0.3 mm) and d′ (*D* = 4 mm) values are in general larger for raw images, but for smaller Al thicknesses differences are small (Figure 4g,i). For the Philips system the d′ (*D* = 0.3 mm) values are consistently larger for clinical images (Figure 4h). However, for d′ (*D* = 4 mm) this is valid only for Al thickness up to 2 mm. For 3 mm Al the d′ (*D* = 4 mm) for raw and clinical images are practically the same, whereas for 4 mm Al and above the d′ (*D* = 4 mm) become larger for the raw images (Figure 4j). For both GE and Philips, the d′ (*D* = 0.3 mm) and d′ (*D* = 4 mm) values increase continuously with increasing dose, but for the clinical images acquired with GE, a plateau is reached at the mAs value selected by the AEC.

***Experiment 4***: For both GE and Philips systems, the MTF 50% values are larger for clinical images and tend to increase with increasing kVp, except for the raw images acquired with the GE system where MTF 50% remains practically constant (Figure 5a,b). For GE the SNR values are larger for clinical images (Figures 5c) whereas for Philips are larger for raw images (Figure 5d). For GE the SNR values are practically constant with increasing kVp for raw images and slightly reduce for clinical images. For Philips the SNR values increase with increasing kVp for clinical images but reduce for raw images. For GE the SDNR values are larger for clinical images (Figure 5e), whereas for Philips the SDNR values are larger for raw images (Figure 5f), but for both systems reduce with kVp.

For GE the d′ (*D* = 0.3 mm) are larger for raw images but at a kVp value of 80 for 0.5‐ and 1‐ mm Al, 90 for 2 and 3 mm Al, and 100 kVp for 4 mm Al and above, the d′ (*D* = 0.3 mm) become slightly larger for clinical images. However, the d′ (*D* = 4 mm) values are always larger for raw images (Figure 5g,i). For Philips the d′ (*D* = 0.3 mm) values are larger for clinical images with a few exceptions (for 50 kV and 4‐, 5‐ and 6‐ mm Al) (Figure 5h). The d′ (*D* = 4 mm) values exhibit a mixed behavior as in general are larger for clinical images for larger kVp and smaller Al thicknesses, whereas for lower kVp and larger Al thicknesses become larger for raw images. For both GE and Philips, the d′ (*D* = 0.3 mm) and d′ (*D* = 4 mm) values decrease with increasing kVp, but the decrease is slightly less for clinical images.

## 4 DISCUSSION

Based on relevant literature, the IAEA methodology^2^ indicates that d′ indices effectively link subjective measurements such as SDNR and MTF to practical, clinical interpretation tasks. Therefore, though the IAEA radiography and mammography phantoms are very simple (compared to commercially available QC phantoms, such as those tested in a previous publication^3^), the d′ indices can be directly linked to the clinical imaging performance. The IAEA methodology advises users to use “for‐processing” (raw) images for the best results and the most advanced analysis. It is emphasized that d′ measurements and calculations need to be performed on raw images that are linearized and offset corrected, as the assumptions used in the calculation of d′ are only valid for raw images. Calculations on clinical images do not have the same broad detectability significance, and d′ cannot be calculated from fully processed images. However, when raw images are not available, the IAEA methodology allows the use of “for‐presentation” (clinical) images for tracking the consistency of post‐processing as well. Thus, ATIA produces the same IQ‐metrics for both raw and clinical images.

The question is whether the results of this study are in line with the above guidelines, that is, verify or question the general notion that d′ values cannot be meaningful when calculated in clinical images. Regarding MTF, which is an IQ metric that exists both in the nominator (squared) and the denominator (square root) of d’ definition,^2^ when looking at Figures 2, 3, 4, 5, it is evident that MTF values are almost always larger in clinical images (with the exception of Chest PA protocol for the GE system), and therefore this is one reason to expect that d′ values should be larger in clinical images for both GE and Philips systems. For raw images the MTF 50% values vary only slightly with protocol selection and dose to the detector for both GE and Philips systems, but the kVp selection has a different effect on the Philips system (MTF 50% values increase with kVp selection) compared to the GE system (MTF 50% values do not change with kVp). For clinical images the MTF 50% values strongly vary with protocol selection (for both GE and Philips), reduce with increased IAK to the detector (strong correlation for GE, weak for Philips), and increase with increased kVp selection (strong correlation for both GE and Philips).

Regarding SNR, this IQ metric does not exist in d’ definition,^2^ and there is only an indirect connection with d′, as the signal (PV) and noise (SDPV) which is measured in four ROIs around the 4 mm Al target of the IAEA phantom and all different thicknesses in the Multi phantom, are also used to calculate SDNR. Though local non‐uniformities do exist, as can be seen in Figures 2, 3, 4, 5, the PV and SDPV of these ROIs are expected to be comparable to the signal and noise of the large red ROI where the normalized noise power spectrum (NNPS) is calculated. Since NNPS (square root) exists only in the denominator of the d’ definition equation,^2^ the larger the noise the smaller the d′ values will be. When looking at Figures 2, 3, 4, 5, it can be seen that SNR variation with image type, protocol, dose, and kVp selections differ much between GE and Philips. For the GE system, SNR values are always larger for clinical images, whereas for the Philips system the opposite is valid, with the differences in SNR values between clinical and raw images being larger for the Philips system. For the GE system, SNR values for clinical images reduce with dose and kVp (weak correlations) but increase with IAK (strong correlation) and kVp (weak correlation) for raw images. On the contrary, for the Philips system SNR for both clinical and raw images increase with IAK (strong correlations), increase with kVp for clinical images but decrease for raw images (strong correlations).

Regarding SDNR, which is an IQ metric that exists (noted as *C*) only in the nominator of the d’ definition equation,^2^ when looking at Figures 2, 3, 4, 5, it is evident than for GE the SDNR values can be larger on clinical or raw images depending on the protocol, whereas for Philips are larger in the raw images for all the protocols tested. However, for both systems differences among clinical and raw images tend to reduce for smaller Al thicknesses. A difference between GE and Philips is that the SDNR values for GE, as well as the d′ (*D* = 0.3 mm) and d′ (*D* = 4 mm) values for all Al thicknesses, increase with dose continuously for raw images though slower for larger IAK values, while for clinical images reach a plateau at the IAK value corresponding to the AEC switch‐off dose. On the other hand, for Philips all these IQ‐metrics increase almost linearly with increasing dose. What is also different between systems is that while for both systems relationship between SDNR, d′ (*D* = 0.3 mm) and d′ (*D* = 4 mm) values for clinical and raw images change with IAK in a similar manner, for Philips the SDNR values are always larger for raw images for all Al thicknesses, while the d′ (*D* = 0.3 mm) values are always for the clinical images, but the d′ (*D* = 4 mm) are larger in clinical images for small thicknesses but larger for raw images for larger Al thicknesses. What is common in both GE and Philips systems is that SDNR, d′ (*D* = 0.3 mm) and d′ (*D* = 4 mm) reduce all with increasing kVp for both clinical and raw images.

According to the general theoretical concepts regarding the relationship of IQ with dose and kVp selection, it is expected that an “ideal” IQ metric should increase with dose because of better statistics and noise reduction (except for doses close to detector saturation) and decrease with kVp because of contrast reduction occurring when the Compton effect outweighs the photoelectric effect. Furthermore, the “ideal” IQ metric should be larger when a detail—from which this IQ metric is deduced—increases in contrast and/or size with respect to the background. Finally, since the “ideal” IQ metric should be related to the diagnostic confidence of the radiologist when interpreting actual patient anatomic images, its value for the clinical images should be the same or larger than that for the respective raw images.

Assuming that the above general concepts regarding an “ideal” IQ metric are valid, it is obvious that MTF 50% and SNR cannot be considered as such a metric, since their variation patterns in raw and clinical images with dose and kVp do not fulfill the above expectations. On the other hand SDNR, d′ (*D* = 0.3 mm) and d′ (*D* = 4 mm), do all increase with increasing dose, reduce with increasing kVp, and reduce with smaller Al‐target thickness for both clinical and raw images. Therefore, the only problem that remains is that they do not follow the same pattern of variation in the two different x‐ray systems, nor they are always better in clinical images, which is not what expected for an “ideal” IQ‐metric. However, this can be attributed to the different approaches that each manufacturer adopts to improve the diagnostic confidence, in an effort to compromise the opposing trends that post‐processing imposes on a digital image. For example, smoothing algorithms reduce the noise but also reduce spatial resolution, while edge‐enhancement algorithms increase the spatial resolution but also increase the noise. Therefore, a single “ideal” IQ metric that can monotonically describe IQ for all clinical diagnostic tasks most probably cannot be defined, since the diagnostic tasks greatly vary in radiology depending on the anatomy imaged and the pathology that is intendent to be imaged. It should also be bear in mind that in the different radiographic examinations different kVp values are used, as a compromise between improved detectability and reduced dose. Indeed, Peteghem et al.^6^ commented that while well‐defined limiting values for the threshold contrast and d’ values are possible in mammography where the imaging task is specific (the detection and characterization of microcalcifications and mass lesions), in general radiology such an approach is not possible, given the extremely broad range of imaging tasks and associated structures and target sizes. They also commented that while in radiology the d’ method (using raw images) offers a useful starting point, feedback from the users may be required to confirm the final clinical operating point. Therefore, for‐presentation (clinical) images covering a range of typical tasks in clinical practice using the respective post‐processing algorithm, will be also required apart from d’ or contrast‐detail curves evaluation.

Considering all the above, it can be said that d′ values may be the best approach of the “ideal” IQ index in clinical images as well. The relatively small differences between the d′ values for raw and clinical images observed for a wide range of Al‐targets thicknesses, seem to question the strong reservations expressed in the IAEA publication against the calculation of d′ values for clinical images,^2^ at least for those DR systems that follow similar post‐processing rationale with either of the two x‐ray systems used in this study.

## 5 CONCLUSION

In this study a modified radiography IAEA phantom (named Multi) was used, which apart from the standard Al target thickness (4 mm), included six more Al squares of 0.5‐, 1‐, 2‐, 3‐, 5‐ and 6‐ mm thickness to simulate additional interpretation tasks. This phantom can be used either as the typical IAEA radiology phantom (employing the 4 mm Al square only), but also as a phantom for research purposes, due to the presence of the step‐wedge like increasing Al‐target thicknesses, which allows to explore the d’ indices variations for a range of imaging interpretation tasks, where the detection difficulty increases with decreasing Al target thickness.

The results showed that the IQ metrics’ values calculated using ATIA and the modified IAEA phantom images may greatly differ between raw and clinical images and between different DR systems of different manufacturers, irrespectively whether only the standard 4 mm Al target or all the different Al‐target thicknesses are considered. This is because their dependence on exposure conditions and post‐processing algorithms do not always follow the same trends. However, the d′ seem to be a reliable IQ metric that can be used for constancy testing to answer the question whether IQ improves or deteriorates when exposure factors change, irrespectively whether raw images or clinical images are used, provided that the same post‐processing protocol is consistently used.

## AUTHOR CONTRIBUTIONS

All authors substantially contributed to the conception or design of the research. Image acquisition and data analysis was made by Ioannis A. Tsalafoutas, and Shady AlKhazzam. All authors contributed to drafting the manuscript or revising it critically for important intellectual content.

## CONFLICT OF INTEREST STATEMENT

The authors declare no conflicts of interest.
